# Factors Associated with the Implementation of Non-Pharmaceutical Interventions for Reducing Coronavirus Disease 2019 (COVID-19): A Systematic Review

**DOI:** 10.3390/ijerph18084274

**Published:** 2021-04-17

**Authors:** Krishna Regmi, Cho Mar Lwin

**Affiliations:** 1 Institute for Health Research, University of Bedfordshire, Luton LU1 3JU, UK; 2 Centre for Medical Education, School of Medicine, University of Dundee, Dundee DD2 4BF, UK; 3 Department of Rheumatology, University of Medicine Mandalay, Mandalay 05024, Myanmar; chomarlwindr@gmail.com

**Keywords:** non-pharmaceutical interventions, social distancing, quarantine, isolation, COVID-19, COVID-19 pandemic, SARS-CoV-2, prevention, control, systematic review

## Abstract

There has been much discussion recently about the importance of implementing non-pharmaceutical interventions (NPIs) to protect the public from coronavirus disease 2019 (COVID-19) infection. Different governments across the world have adopted NPIs (e.g., social distancing, quarantine, isolation, lockdowns, curfews, travel restrictions, closures of schools and colleges). Two fundamental strategies, namely a strict containment strategy—also called suppression strategy—and a mitigation strategy have been adopted in different countries, mainly to reduce the reproduction number (R_0_) to below one and hence to reduce case numbers to low levels or eliminate human-to-human transmission, as well as to use NPIs to interrupt transmission completely and to reduce the health impact of epidemics, respectively. However, the adoption of these NPI strategies is varied and the factors impacting NPI are inconsistent and unclear. This study, therefore, aimed to review the factors associated with the implementation of NPIs (social distancing, social isolation and quarantine) for reducing COVID-19. Following PRISMA guidelines, we searched for published and unpublished studies, undertaking a systematic search of: MEDLINE, EMBASE, Allied and Complementary Medicine, COVID-19 Research, WHO database on COVID-19, and Google Scholar. Thirty-three studies were included in the study. Seven descriptive themes emerged on enablers and barriers to NPIs: the positive impact of NPIs, effective public health interventions, positive change in people’s behaviour and concerns about COVID-19, the role of mass media, physical and psychological impacts, and ethnicity/age associated with COVID-19. This study has highlighted that the effectiveness of NPIs in isolation is likely to be limited, therefore, a combination of multiple measures e.g., SD, isolation and quarantine, and workplace distancing appeared more effective in reducing COVID-19. Studies suggest that targeted approaches alongside social distancing might be the way forward, and more acceptable. Further research to promote country- and context-specific adoption of NPIs to deliver public health measures is needed. Studies comparing the effectiveness of interventions and strategies will help provide more evidence for future pandemics.

## 1. Introduction

Coronavirus disease 2019 (COVID-19; caused by severe acute respiratory syndrome coronavirus 2 (SARS-CoV-2)), emerged in Wuhan, China in December 2019, and is currently the greatest public health challenge. At the time of writing (9 April 2021), the WHO COVID-19 Dashboard reports that this virus has already affected 223 countries and territories with approximately 133,552,774 confirmed cases and 2,894,295 deaths; a fatality rate of 2.17% [1]. Recently there has been much discussion about the importance of implementing NPIs to protect the public from COVID-19 infection. To control the virus, different governments across the world have adopted different NPIs or measures (e.g., social distancing, quarantine, isolation, lockdowns, curfews, travel restrictions, closures of schools and colleges) [2,3]. On the basis of the feasibility of blocking virus transmission, disease estimation and severity, socio-economic and political strategies, public willingness and acceptability including government capacities and capabilities, two major intervention strategies—the strict containment strategy (also called suppression strategy) and the mitigation strategy—have been adopted in different countries [2,4,5]. A strict containment strategy involves being “proactive in detecting and managing cases, tracing and isolating close contacts and strictly restricting or controlling population movement—these are mostly adopted in China, Japan, Singapore and Thailand”. The purpose here is to reduce the reproduction number (the average number of secondary cases each case generates), R, to below one and hence to reduce case numbers to low levels, whereas a mitigation strategy incorporates the treatment of severe cases and utilising NPIs, rather than optimising the detection and management of each case and close contacts and this was mostly adopted in Europe (UK, Italy, France) and the United States [2,5,6]. A mitigation strategy aims to not interrupt transmission completely, but to reduce the health impact of an epidemic and such a strategy was adopted by some US cities in 1918, and by the world more generally in the 1957, 1968 and 2009 influenza pandemics [5]. Social distancing as a form of NPI or physical intervention has been practised in several countries, including the UK, USA and some EU countries, to reduce transmission and associated illness or deaths [7].

Infectious disease spread surveillance and predictions inform pharmaceutical interventions (also called disease mitigation strategies), using vaccines or medicines, as well as NPIs (also called community mitigation strategies) using face coverings, social or physical distancing, isolation of ill persons, quarantine of exposed persons, contact tracing, travel restrictions, school and workplace closures, and cancellation of mass gatherings [8,9]. NPIs are public health actions to prevent and/or control SARS-CoV-2 transmission in the community by slowing the spread of illnesses. Generally, NPIs are considered the best way of controlling a pandemic when the general population has little or no immunity against them and the vaccines are subject to no or limited availability [10,11].

In relation to COVID-19, the meanings and interpretations of the term social distancing vary, as some consider it a policy, strategy or approach to flatten the curve by simply increasing physical distance [7,12,13,14]. In reality, it also includes hand hygiene, use of face masks and coughing etiquette. We considered social distancing (SD) as a set of NPIs to prevent the spread of COVID-19 by maintaining physical distance between people and reducing the frequency of close contact between people [7,13,15]. In general terms, the working mechanism is increasing the distance between individuals to reduce the likelihood of viral materials being able to travel from the airway of an infected individual to that of an as yet uninfected individual by means of aerosolised droplets produced by coughing or sneezing. With a wide enough distance between individuals, the ejected viral material does not have enough time to stay airborne to be inhaled before it settles on the ground or other surfaces where, in time, it dies or is wiped off [7,13]. This mechanism is different from face masks, which are supposed to work by forming a barrier that greatly cuts down the travel distance of viral material in exhaled air, both at the source and also at the receiver end (if both wear masks) [14]. This study focused mainly on three major NPIs—SD, isolation and quarantine—and the associated impacts on reducing COVID-19 transmission. Isolation of cases is the separation of infected from non-infected cases to provide care and support in the hospital or at home, and quarantine is the restriction of exposure to disease when not ill [16]. The WHO also recommends SD (physical distancing), isolation and quarantine as major NPIs for reducing transmission of COVID-19 [17].

Since the COVID-19 pandemic, the use of social media (SM) has increased greatly e.g., there was a 61% increase in usage as people use the platforms to stay connected with family, friends, and colleagues, a 70% increase in WhatsApp, Facebook and Instagram users and views on live streams doubled [18,19]. In fact, SM during COVID-19 has contributed positively to informing the public about the perceptions of risks and mitigation [20] as well as changing their appropriate behaviours under NPIs, e.g., SD mandates [21]. SM exposure has also had an indirect positive effect on preventive behaviours, which are mediated by fear and anger [22,23]. SM’s explosive growth can be at least partially explained by the worldwide SD directives and lockdowns [19]. SM offers opportunities for both experts and the general public to quickly spread information to a large number of individuals [20]. Health professionals played a critically important role as trusted sources on SM, not only to support the spread of new information but also to address people’s worries and concerns. In that sense, SM has been proven as an asset to developing effective risk communication strategies and response [19]. The unfolding of the COVID-19 pandemic has demonstrated how the spread of misinformation, amplified on SM and other digital platforms, is proving to be as much a threat to global public health as the virus itself, as it continues to undermine the global response and jeopardises measures to control the pandemic [24,25]. Despite clear evidence that NPIs, mainly wearing masks, can reduce transmission of COVID-19, the acceptability of and adherence to wearing masks varies greatly. In the United States, face mask wearing has become more of a political issue than a fact-based intervention, and thus the use of face masks varies widely among populations. In other communities and countries, mask wearing is seen as a reasonable strategy, and face masks are commonly used by the public [20,26]. The variation in acceptability and willingness for individuals to respond to expert opinions creates significant challenges for health education among patients [22,26].

Evidence indicates that in England, Black, Asian and Minority Ethnic (BAME) groups recorded higher mortality, ranging from 1.5 (Asian population) to 7.3 (Black Caribbean population) times greater than white individuals [27], and 1:10 reported infections were among health professionals, including medical doctors and nurses. In the USA, meanwhile, the rate for African Americans was 2.4–2.7 times more than white individuals. Deaths are not consistent across these groups; however, it has a clear impact by income—hitting hardest the most deprived populations in low- and middle-income as well as in high-income countries (e.g., having low income or lacking health insurance) [28]. Several factors, e.g., higher rates of comorbidities (such as diabetes and renal conditions), co-habiting in inter-generational family units, employment in frontline roles, socioeconomic status, and lower health-seeking behaviour may contribute to this increased risk [29,30,31].

When we look at evidence focusing on BAME/African Americans in connection with NPIs, we did not find any specific data or evidence that connect with NPIs, but there are a number of possible reasons reported in the literature. Firstly, staff from ethnic backgrounds have had more negative experiences related to discrimination and safety in the workplace during COVID-19 [32]. Secondly, ethnic backgrounds have been less likely to have had adequate PPE (e.g., facemasks, eye protection) [32]. Thirdly, in the UK, for example, higher proportions of Pakistani (20%) and Indian (20%) key workers compared with white counterparts have reported that their safety complaints have been ignored [32,33]. Fourthly, ethnic groups were more likely to be working outside their home during the lockdown period and they were less likely to be given PPE and more likely to be given tasks that exposed them to the coronavirus [32,33]. Fifth, people from ethnic backgrounds are more likely to live in an overcrowded household with several generations or in a household of multiple occupations [34,35,36], which have been shown to increase the risk of COVID-19 infection and mortality [32,37]. In addition to this, a major failure in the British and American cases has been the inability to set up a properly functioning “test, trace and isolate” system [32]. Finally, there has also been little guidance on how to develop culturally competent preventive public health and risk reduction recommendations [38].

Similarly, evidence suggests that the number of cases reported is increasingly due to improved diagnostic capacity for COVID-19; for example, antigen rapid diagnostic tests (Ag RDTs) are faster and cheaper than laboratory-based tests which often provide results within 15–30 min. Most importantly, these tests are performed even in countries where they do not have extensive laboratory facilities or trained health workers. From a public health point of view, these enable countries to increase the pace of testing, tracing and treating people for COVID-19 at the point of care, particularly in areas with under-resourced healthcare systems [39,40]. Still, low- and middle-income countries are particularly vulnerable to the surging pandemic. Many have no domestic capacity for manufacturing diagnostic tests and rely heavily on imports [41]. While molecular tests (PCR) are mainly laboratory-based, infrastructure and trained personnel are relied on to conduct them [24,39].

A scoping search of MEDLINE in January 2021 for publications entered by the end of December 2021 used the following terms: ((“COVID-19” OR “SARS-CoV-2”) AND (“systematic review” OR “literature search” OR “meta-analysis” OR “evidence synthesis”) AND (“social distancing” OR “isolation” OR “quarantine”)). Though some studies reported on COVID-19 from different countries including the UK, USA and China, most were not reviewed or synthesised systematically. Those revealed were mostly on COVID-19 epidemiology [42,43], effects of school closure [44], quarantine [45,46] and mathematical modelling [5,9,47,48,49,50,51] and SD [52], based on two previous reviews [53,54] on influenza.

Recently, Chu and colleagues [55] conducted a systematic review including physical distancing, focusing more on face masks and eye protection, to examine the optimum distance for avoiding person-to-person transmissions. Though this study identified 172 studies across 16 countries and six continents, none of the studies were randomised controlled trials (RCTs), therefore, their findings might suffer from both recall and measurement biases. Similarly, Jefferson et al. captured 67 studies including RCTs and observational studies, to examine the role of physical interventions in interrupting or reducing the spread of respiratory viruses, but found no strong evidence of SD [55]. Though some studies observed that combined NPIs e.g., SD, isolation and quarantine and workplace distancing, appeared effective in reducing COVID-19, these still reported some challenges, e.g., societal disruption, social isolation/rejection, mental stress and psychological trauma, lack of tests and testing facilities, poor contact tracing and lack of surveillance [31,56]. None of the systematic reviews examined these factors that impact NPIs for systematically reducing COVID-19 transmission. This systematic review, therefore, aimed to identify the factors that have been reported in the medical literature associated with the implementation of NPIs for reducing transmission of COVID-19.

### Research Question (RQ)

What are the factors associated with the implementation of non-pharmaceutical interventions (social distancing, social isolation and quarantine) for reducing coronavirus disease 2019 (COVID-19)?

## 2. Materials and Methods

The protocol was registered with PROSPERO (registration number: CRD42020207338), and a detailed pre-registered protocol was also published elsewhere [57]. We conducted a systematic review (SR), using systematic, explicit and accountable methods to minimise biases and random errors, in order to answer a specific RQ [58]. The main reasons for choosing an SR in this study are as follows: firstly, while doing a scoping research of MEDLINE on the topic, we did not come up with any SRs that examined factors associated with NPIs systematically. Secondly, the methods and procedures used in SRs would provide an empirical basis for guiding decisions through summarising the evidence ([59], p. 157). Thirdly, SRs can highlight non-evidence-based practice and provide a sound basis for both policy and practice. Finally, this study followed a published protocol that might reduce publication bias [59]. It is, however, equally important that SRs are not immune from bias. As Guyatt and Rennie ([59], p. 180) argue for SRs, a clear set of rules is used to search for studies, and then in determining which studies will be included in or excluded from the analysis, there is an element of subjectivity in setting these criteria, as well as in the conclusions drawn, so we cannot say that SRs are entirely objective. However, because all of these decisions are specified clearly, the mechanisms are transparent ([60], p. xxiii).

### 2.1. Criteria for Considering Studies for the Study

Inclusion Criteria

1. Types of participants: all studies that involve human subjects of any age or gender, including ethnic (Black, Asian, White) and healthcare worker (medical doctors, nurses, allied healthcare professions) groups.
2. Types of intervention: research describing three major NPIs, e.g., social distance, isolation and quarantine, focusing only on COVID-19/SARS-CoV-2.
3. Types of outcome measure: primary outcomes include: COVID-19; reducing the risk of transmission/infection of COVID-19; secondary outcomes include changes in social behaviour, for example, SD by avoiding crowds, restricting movements, isolating ill patients and quarantine of exposed people.
4. Types of studies: no study design filter is added. To measure the impact of NPIs, this review considered all studies evaluating the effectiveness of NPIs relating to reducing the risk of transmission/infection of COVID-19. We included both RCTs and non-RCTs, for example, cross-sectional, survey, case-control, RCTs and observational studies (retrospective or prospective) including preprint engines such as medRxiv, bioRxiv, Litcovid and SSRN for unpublished studies on COVID-19, given the lags in publication.
5. Study period: December 2019 to March 2021.

Exclusion Criteria

1. Articles published in narrative review, modelling studies, opinions, letters, news, editorials, perspectives, commentaries and any other publications lacking primary data, including grey literature.
2. Studies containing duplicate datasets.

### 2.2. Search Strategy

The electronic databases MEDLINE, EMBASE, Allied and Complementary Medicine, COVID-19 Research, the WHO database on COVID-19 and Google Scholar on COVID-19 from December 2019 to March 2021, with the last search conducted on 12 March 2021, to contemplate the recent pandemic. We searched for articles using the following search strategy (Table 1).

We utilised two citation-based search methods of the “related articles”, i.e., the best match and most recent features in PubMed, to identify additional papers, and the first 20 linked articles were screened. Searches were also supplemented by reviewing the reference lists (‘reference of references”) of selected articles to find other relevant papers and any relevant reviews identified in the literature reviews. The literature search strategy was developed in collaboration with departmental subject librarians from authors’ universities, who were experienced in SRs, and subsequently refined for comprehensiveness. We also contacted six study authors to identify additional studies.

### 2.3. Selection of Studies

**Figure 1:**
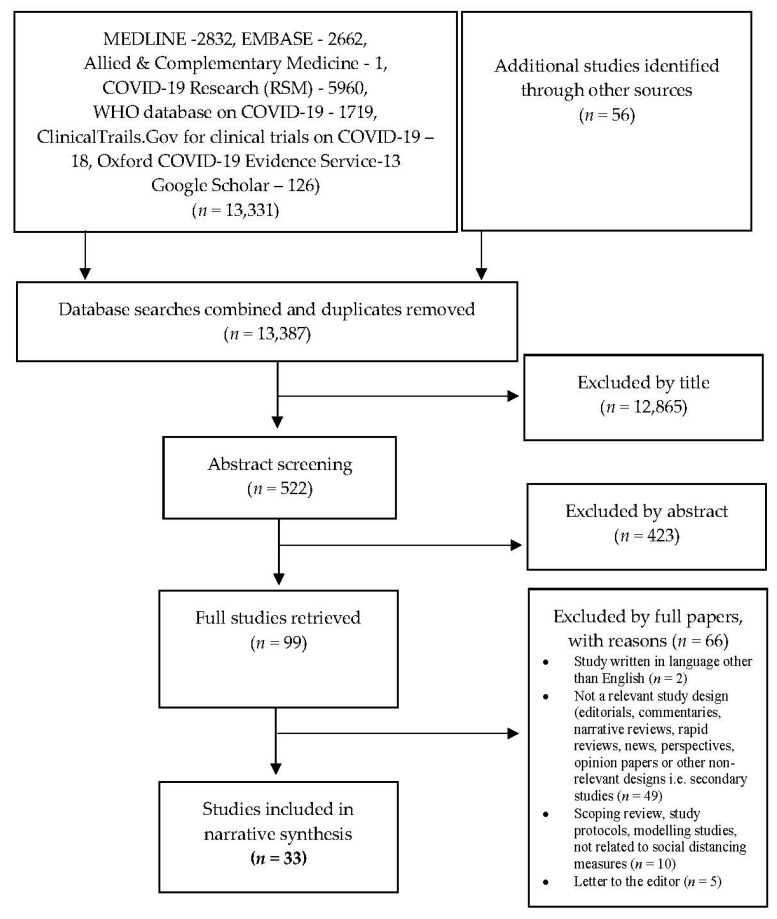
PRISMA flow diagram to show results of searches.

The citations identified through the searches were imported into Mendeley Reference Manager (https://www.mendeley.com/ (accessed on 10 April 2021)). All studies emerging from the databases were screened twice: (i) screening of titles and abstracts against inclusion criteria, and (ii) review of the full text. We used the standard PRISMA flow diagram to provide the process of study selection [61] (Figure 1).

### 2.4. Data Extraction, Analysis and Synthesis

**Figure 2:**
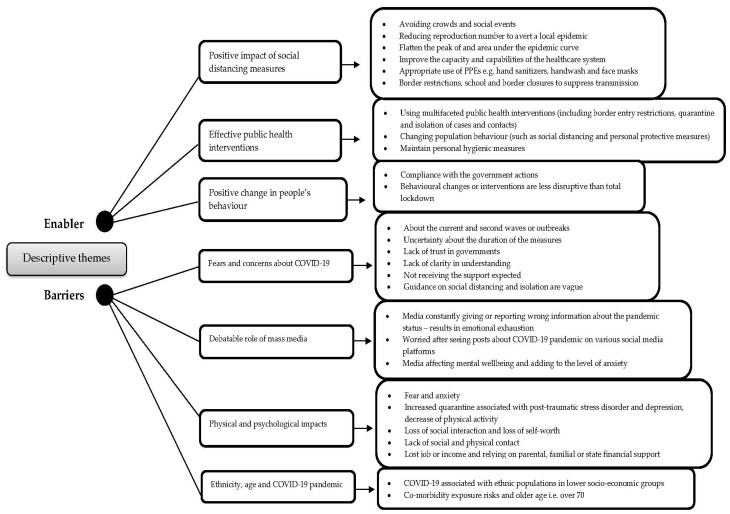
Themes identified across studies.

Types of data from the included individual study are not suitable (e.g., dichotomous, continuous) for effective measure due to variation across studies (heterogeneity), and outcomes appeared rather diverse due to degree of difference or variance among samples, groups and populations, hence statistical combinations of results (meta-analysis) are not possible [60]. Therefore, results are summarised using narrative synthesis and tabular form using thematic analysis (Figure 2, Table S1). Once articles were pulled, we removed duplicate documents from the different databases. Titles, keywords and abstracts of all downloaded citations were screened and paper copies of those meeting our selection criteria were retrieved. Two reviewers (KR, CML) extracted data independently using a data extraction sheet. Data were extracted using the following summary data: sample characteristics, i.e., study aim, study location, study design, sample size, and appraisal checklist(s) and the overall reviewer comments. The two reviewers scored the final set of articles independently and then averaged the score. Themes were ordered according to the number of studies in which they were identified. Thematic analysis/synthesis was used to identify the important or recurrent themes and the findings summarised thematically [62]. In this process, we followed Thomas and Harden’s [62] approach. First, a read and re-read of the studies to develop an initial level of codes reflecting various ideas or concepts within the data was carried out. Such a technique allows one to not only translate the concepts across studies but also to develop further codes through adding, merging or altering codes emerging from the data. Second, we identified some similarities and differences in the codes and then we grouped them (into the cluster) based on the similarities and differences, which ultimately helped us to create descriptive themes. The coding process and development of descriptive themes were discussed among the authors.

### 2.5. Quality Appraisal (Risk of Bias)

We assessed the study quality using the Joanna Briggs Institute (JBI) checklists, instead of the Newcastle-Ottawa Scale (NOS) mentioned in the protocol, for the quality assessment of included non-RCT articles for the cross-sectional survey [63], qualitative [64], cohort [63] and case-control [63] studies to assess the methodological quality of a study and to determine the extent to which a study has addressed the possibility of bias in its design, conduct and analysis. We recently noticed that the NOS scale has never been validated and it measures a whole range of things that are not all to do with the reliability of the findings obtained. The JBI checklist included the four-item checklists with standardised questions, i.e., yes, no, unclear and not applicable. We consider ≥7 points as good quality, 6 points as fair quality and ≤5 points as poor quality. The results have been used to inform the synthesis and interpretation of the findings. To facilitate comparison of appraisal processes, all reviewers recorded the rationale for inclusion or exclusion, and discrepancies were discussed and resolved by consensus. We also used the assessment to comment on the general quality of included studies across these items. The quality of each study was assessed by two reviewers (KR, CML) who independently screened the papers in two steps, i.e., first abstracts and then full texts and extracted data.

## 3. Results

Our broad database searches identified 13,387 records (13,331 records identified through databases and 56 records identified through additional sources) (Figure 1). After title and abstract screening, 99 papers were retrieved for full paper review. After the full paper screening, 33 studies met our full review inclusion criteria [65,66,67,68,69,70,71,72,73,74,75,76,77,78,79,80,81,82,83,84,85,86,87,88,89,90,91,92,93,94,95,96,97]. As reported in Figure 1, the first screen based on the inclusion criteria saw a large number of publications not being included. We observed that our systematic search yielded a large number of duplicate references, for two main reasons: first, COVID-19 Research (Royal Society of Medicine (RSM)) database provider Dialog is made up of the same content from MEDLINE, EMBASE and Allied and Complementary Medicine. Second, both COVID-19 Research (RSM) and the WHO database on COVID-19 retrieved similar studies. In addition to this, the majority of the studies were excluded by title, as although databases appropriately picked the subject, e.g., COVID-19, most of them were not primary studies.

### 3.1. Study Characteristics

We found 33 observational studies (with 116,897 participants). Of the 33, 26 (78.78%) were quantitative cross-sectional surveys [65,66,67,68,69,70,71,73,74,76,77,79,81,82,84,85,86,87,89,91,92,93,94,95,96,97], four (9.09%) were qualitative studies [75,78,88,90], two were cohort studies [72,83] and one was a case-control study [80]. All studies recruited participants online. Studies were conducted in 17 countries including the UK, USA, Germany, China and Italy. These studies are summarised in Table 2.

### 3.2. Risk of Bias

A summary of results from the JBI appraisal [63,64] quality appraisal of the four-item checklists, i.e., yes, no, unclear and not applicable, can be found in Table 2, and the critical appraisal criteria can be found in File S1. There was a fair degree of methodological heterogeneity across the 33 studies. The majority were quantitative (26/33) which mostly used questionnaire surveys, followed by qualitative (4/33), cohort (2/33) and case-control (1/33) studies. The quality of the 33 studies was generally poor (14/33) when tested against the appraisal criteria. These were poor in providing methodological details around recruitment strategies and sampling. Studies from quantitative designs mostly failed to provide clear inclusion and exclusion criteria, did not discuss exposure measures or potential confounding factors and also failed to provide baseline characteristics. Similarly, studies of qualitative design failed to meet the criteria of reflexivity and these studies were not appropriately linked or discussed with researchers’ cultural and theoretical orientations, as well as not adequately addressing the relationship between the researchers and the study participants at all. Confounding factors, strategies to minimise and follow-up and outcome assessed were not standard, valid and reliable for cohort and case-control studies. More than half of the included studies (19/33, 13 quantitative, three qualitative, one case-control and one cohort) met the majority of the relevant quality criteria. Most of these studies had clear research designs where they appropriately discussed research methods and procedures (Table 2).

### 3.3. Synthesis of Results

In general, these studies cover at least one of two areas: (i) positive impacts (enablers) and (ii) specific barriers to control or reduce transmission of COVID-19. Eight important themes under two broad descriptive themes emerged (Figure 2). The relative contribution of each study to the synthesis is in Table S1.

Enablers

- Theme 1. Positive impact of SD measures.
- Theme 2. Effective public health interventions.
- Theme 3. Positive changes in people’s behaviour.

Barriers

- Theme 4. Fears and concerns about COVID-19.
- Theme 5. Debatable role of mass media.
- Theme 6. Physical and psychological impacts.
- Theme 7. Ethnicity, age and COVID-19 pandemic.

### 3.4. Enablers

#### 3.4.1. Theme 1. Positive Impact of SD Measures

Fourteen out of 33 studies identified some positive impacts of different NPIs used to reduce transmission of COVID-19 [65,66,67,68,70,71,77,90,91,92,93,94,95,96]. The commonest NPIs were avoiding crowds, border restrictions, isolating in hospital, appropriate use of PPE and working from home primarily to reduce the effective reproduction number of SARS-CoV-2 (R_0_, secondary transmission).

One study has highlighted that:

Without strengthening SDMs, local infections are likely to continue occurring, given that the effective reproduction number (R_0_) is approximately 1 or slightly higher. Travel measures and testing, tracing, and treating efforts are particularly important in maintaining suppression, although these measures will be increasingly difficult to implement as case numbers increase [66].

Similarly, other studies further added that if the basic reproduction number of COVID-19 in Hong Kong, the UK and the US exceeds 2 (it was 2.2 in Wuhan), we would need a >44% reduction in COVID-19 transmission to completely avert a local epidemic. A reduction of this magnitude could, however, substantially flatten the peak of and area under the epidemic curve, thus reducing the risk of exceeding healthcare system capacity, potentially saving many lives, especially older adults [65,66,68,88,89,93].

Studies reported that quarantine, and school and border closure have been the most effective means of suppressing transmission [66,92]. The commonest factors associated with NPIs success are supporting governmental measures for SD and isolation by avoiding crowds, closure of public places, hand hygiene and individuals’ adherence to country-specific mitigation measures [89,94,96].

#### 3.4.2. Theme 2. Effective Public Health Interventions

Nine of 33 studies reported the importance of public health interventions for COVID-19 [65,66,67,68,69,88,94,95,96]. Several studies perceived handwashing with soap and avoiding crowds and social events as the most effective measures [65,71,88,89,94]. Several studies from different parts of the world reported that multifaceted public health interventions including personal protective equipment (PPE, e.g., facemasks, eye protection), have been successful as the virus spreads through multiple channels, e.g., touching, sneezing.

The extracts below illustrates this:

The package of public health interventions (including border entry restrictions, quarantine and isolation of cases and contacts, and population behaviour changes, such as social distancing and personal protective measures) that Hong Kong has implemented since late January 2020, is associated with reduced spread of COVID-19 [66].
The study participants reported frequent use of sanitisers, hand wash, and masks during the past week. This indicates participants’ increasing concern towards personal hygienic measures. Awareness about COVID-19 is reflected in behaviour and attitude as most participants agreed with social distancing, avoiding travel, self-quarantine and adequate hygiene [68].

#### 3.4.3. Theme 3. Positive Changes in People’s Behaviour

Four studies (of 33) reported SDMs influenced people’s behaviour [65,66,70,77]. Atchison et al. [65] reported that part of the success in early February 2020 was changing people’s behaviour to comply with government actions.

The extracts below illustrate this:

Social distancing and population behavioural changes with social and economic impacts less disruptive than total lockdown can meaningfully control COVID-19. Control measures and changes in population behaviour coincided with a substantial reduction in influenza transmission in early February 2020. This observation suggests the same measures would also have affected COVID-19 transmission in the community, because of some similarities, as well as differences, in the modes of transmission of influenza and COVID-19 [66].
Avoiding close contact, washing hands and wearing facial masks were considered the most protective measures [77].
Hand hygiene is a major element in the prevention of COVID-19 and other infectious disease [90].

### 3.5. Barriers

#### 3.5.1. Theme 4. Fears and Concerns about COVID-19

Twelve of 33 studies reported some concerns about the current pandemic and a possible second wave of COVID-19 [65,66,67,68,72,75,81,88,90,91,92,97]. Therefore, many countries tightened the restrictions. The commonest associated factors were: (i) uncertainty about the duration of measures, increasing number of cases and deaths and their ability to cope longer-term [88,90], (ii) form and frequency of contacts of individuals with COVID-19 [72,75,83] and (iii) lack of trust in public health officials and governments due to lack of clarity about the information on infection and what SDMs are effective against COVID-19 [68,76,81,82,86,90,97].

The extracts below illustrate this:

Overall, 77.4% (1640/2108) of respondents reported being worried about COVID-19 in the UK. For those not previously testing positive for COVID-19, 47.5% (979/2108) believed it was likely they would be infected at some point in the future under the UK Government’s preventive measures. If infected, just over half (56.9%) would expect to be moderately/severely affected (e.g., may need self-care and rest in bed) [65].
People are very worried and are not willing to go to health facilities even if they have general problems. They used to contact health personnel through phone calls, but they were not willing to visit any health centres due to fear of getting COVID-19 from health workers. Fear of transmission was pervasive among health workers as well [90].
The analysis of contact characteristics showed that the incidence rate of close contacts who lived in the same residence was 17.9%, significantly higher than those of other groups with different forms of contact. The incidence rate of relatives was 10.7%, with the highest risk of infection among all relationship groups. The results showed that the closer the contact distance and the higher the frequency of contact, the greater the risk of infection [72].
Most participants felt that guidance on social distancing and isolation had been generally unclear, although some described how it had “become clearer”. Many participants exhibited lack of trust in government or in the media [88].
One study has highlighted the implication of health systems:
The weak infrastructure, under-resourced health system, widespread of the illiteracy and social practices will negatively influence the spread of the COVID-19 and response towards its prevention [89].

#### 3.5.2. Theme 5. Debatable Role of Mass Media

Three of 33 studies identified this as a barrier [69,90,94]. These studies found that rumours on SM and electronic and print media about SDMs (isolation, self-quarantine), and total restriction of travel (curfew) were associated with negative impacts on mental health as they constantly depict the pandemic and deaths related to it. Therefore, people become angry, restless, worried, have difficulty coping, and feel emotionally exhausted [69].

One study observed that:

Approximately 28% of people report sleep difficulties. More than two-thirds of participants reported themselves worried after seeing posts about COVID-19 on social media [69].
Approximately 46% of participants reported worry regarding discussion of COVID-19 in news channels and print media. This indicates a significant proportion of survey participants, despite having adequate awareness about coronavirus infection, are largely influenced by media information. Media influences mental wellbeing and adds to anxiety levels [69].

#### 3.5.3. Theme 6. Physical and Psychological Impacts

Eleven of the 33 studies identified these barriers [69,74,78,82,85,86,87,88,90,91,94]. The commonest associated factors were: anxiety [85,87,91], increased time in quarantine associated with post-traumatic stress disorder, depression [74,87,91], decrease in physical activity [82,94], loss of social interaction, and emotional and psychological distress [88].

The extracts below illustrate this:

The mandated lack of social and, especially, physical contact with family members were identified as particularly difficult. Confinement at home and work, being unable to see friends, being unable to shop for basic necessities of everyday life, and being unable to purchase thermometers and prescribed medications enhanced their feeling of distance from the outside world [71].
All participants felt that the social distancing and isolation policies had had significant social and psychological impacts on their lives and the central theme was loss […]. These emotional and psychological losses were particularly acute for those living in more urban, densely populated cities like London or Birmingham. They were also especially evident amongst those in low-paid or precarious occupations, who had either lost their job or income or were now relying on parental, familial or state financial support as a result of the pandemic [88].
During the COVID-19 pandemic parents emerged with various type of emotional problems, to the extent that some parents experienced symptoms of anxiety (6.6%) and depression (21.7%) which included washing their hands frequently and findings themselves preoccupied with physical discomfort [91].

#### 3.5.4. Theme 7. Ethnicity, Age and COVID-19 Pandemic

We found 13 studies that reported, ethnicity, age and the COVID-19 pandemic [65,67,70,73,77,78,79,80,84,88,91,92,95]. These studies found that COVID-19 was often associated with people from BAME populations in lower socio-economic groups, employment in a lower band/category, other comorbidities, exposure risks and older age.

The extracts below illustrate this:

More disadvantaged backgrounds were less likely to be able to work from home or self-isolate if needed, suggesting structural barriers to adopting preventive behaviours in these groups. The most economically disadvantaged in society are less able to comply with certain NPIs, likely partly due to their financial situation [65].
Adoption of SDMs was almost twice as likely in people over 70 compared to adults aged 18 to 34. Notably, those that were single were less likely to practise social distancing. There was a strong association between socioeconomic deprivation and ability to adopt NPIs [65,92].

## 4. Discussion

This study was undertaken to synthesise the evidence concerning NPIs to reduce COVID-19 transmission. In this study, we found major factors, enablers or barriers, impacting NPIs, emphasising the positive roles of NPIs, public health interventions, behaviour changes, people’s fears and concerns, myths, stigma and physical and psychological impact, including the debatable role of media. Similar issues have been documented in the literature [94,98,99]. The purpose of NPIs is to inhibit the intensity of transmission (R_0_) to reduce R_0_ to <1 or “contain the outbreak within a manageable duration” [100]. The ultimate strategy is to slow down or curb the spread of the overall disease burden—morbidity, severity, fatality, health complications and socio-economic consequences—and reduce the impact on health services. Anxiety or worries about the duration of quarantine have been highlighted in this study. A similar issue has also been reported in previous studies. Sjödin et al. ([101], p. 2), for example, based on the experience of the COVID-19 outbreak in Italy, discussed that for an average household of three persons, around 30 days will be a sufficient length under conditions of near-complete community quarantine adherence. With only medium adherence a duration of 54 days would be necessary, assuming 10% of infections are asymptomatic. In this case, seven secondary cases would be expected in a population of 5000, or 70 secondary infections in a population of 50,000, assuming 10% of infections are asymptomatic.

This study found that NPIs were effective only if integrated with enhanced personal hygiene, environmental sanitation and adequate and appropriate use of PPE (use of masks, handwashing and coughing etiquette). Early diagnosis and prompt management of confirmed cases by isolating (physical distance), timely follow-up and quarantine recommendations (10–14 days) for close contacts of a case constitute the CORE of COVID-19 control. This finding is consistent with the conclusions of a study conducted by Chu et al. [55] i.e., “The risk for infection is highly dependent on distance to the individual infected and the type of face mask and eye protection worn. From a policy and public health perspective, current policies of at least 1 m physical distancing seem to be strongly associated with a large protective effect, and distances of 2 m could be more effective”. Therefore, it is recommended to think of the three Cs: closed spaces, crowded places and close contacts. The ECDC report ([102], p. 3) highlighted that: “The success of social distancing measures (NPIs) that are implemented over an extended period may depend upon ensuring that people maintain social contact—from a distance—with friends, family and colleagues” as well as the strictness of quarantine adherence, household size and highest rates of compliance [100]. Similar issues have also been reported by the findings of our study.

However, very little was known on our specific research question on the extent and the factors impacting NPIs in reducing transmission of COVID-19 nationally and globally, as we are aware of no published systematic reviews report on this subject, or commentaries examining the factors associating NPIs and COVID-19. There are, however, some rapid reviews, summaries and mathematical modelling studies covering COVID-19, in China, South Korea, the UK, the USA and other countries, but the literature has not been systematically reviewed or synthesised. Similarly, the implementation of social distance differs by country due to the wide range of predictors associated with this measure, some far stricter than the UK, with Sweden at the opposite end of the spectrum. Therefore, it is difficult to assess which specific NPIs or measures would have a higher impact on the effects of SD to reduce transmission [103]. Recently, in late December 2020, a novel SARS-CoV-2 variant, VOC 202012/01, emerged in England, UK, and appeared to be rapidly spreading towards fixation which is associated with an increase in the estimated R0 as well as increased risk of death [104]. Though the mechanism of the variant is unknown, the most important fact to reduce the emergence of new mutants is to reduce the spread of the virus, where appropriate implementation of NPIs is still relevant.

Lack of awareness and misconceptions about COVID-19 and the physical and psychological impacts due to lockdowns have been reported in the included studies, and similar findings were reported from previous studies [16,101]. Therefore, there is a need to intensify awareness, education and campaigns targeting general and specific spheres of populations, and to utilise internet-based information with the use of social influencers, education and counselling (IEC) strategies to correct these misconceptions and provide support by different stakeholders (governments, NGOs, charities, national volunteers, community support groups). Increased media coverage would be one key strategy to make NPIs successful. The effectiveness of media and messaging can be influenced by the credibility of the messenger and the content and context of the message [105,106]. Similarly, despite the alarming rate of COVID-19 transmission, the general public are not in full compliance with pandemic guidelines [21]. Policy-makers and public health officials should also be strategic in communicating pandemic-related messages not only considering the psychological characteristics of different groups [22], but also ensuring policy and recommendations are relevant to young people in a climate of misinformation, scepticism and fear [24].

In this study, we also found compliance has been one important factor, but it was not easy for securing public compliance in liberal democratic societies. Similarly, the approach in authoritarian regimes, e.g., China, would likely be unacceptable in other parts of the world. Related to this is how long restrictive measures can be tolerated, which lacks solid evidence [100,102]. Moreover, SD has become a highly charged topic creating much debate among politicians, economists, and medical and public health professionals. The likelihood is that COVID-19 will become endemic, which suggests long-term behavioural adjustments as reported in our study [94]. Similarly, we argued that SD is not part of the culture in either developed or developing countries, for different reasons [107]. In developing countries, it is more related to population density, crowding, workplace conditions etc., such as overcrowding in public transport. In developed countries such as Switzerland, people were still engaging in Swiss kiss as late as 20 March, when COVID-19 was already peaking. Similarly, our study found a relationship between SD and economic aspects: poverty, living in slums, etc. in developing countries. A similar issue has also been reported in the previous study [108]. Therefore, there is a need to completely change the way the economy, businesses and life are organised to protect vulnerable groups such as the homeless, disabled, undocumented migrant workers and inmates. Similarly, home life should be looked at, as evidence suggests we need to change the way we interact at home, for example, with vulnerable family members—elderly, pregnant or immunocompromised due to chronic disease or protracted illnesses—at least until the pandemic is over, e.g., curbing the possibility of transferring the disease to the elderly.

Moreover, we found that due to lockdown, people lost their jobs affecting their income, and suffered job insecurity in general, but it disproportionately affected the most disadvantaged populations. These findings are consistent with previous studies [27,29,109]. Finally, this study along with other evidence suggests that our health systems have not been proactive enough to cope with the current pandemic [70,91]. We argue that public health has failed to convince politicians to take rapid action on prevention of spread or prepare for necessary treatment arrangements. These findings are consistent with those of Pollock et al. [110] and Regmi et al. [111] and they found that the “structure and capacity of our depleted healthcare system are now largely driving the response to this epidemic” and most likely “it will continue to do so until services that support local communicable disease control are rebuilt and reintegrated”.

This study adds to the literature on highlighting the major enablers and barriers of SDM in controlling COVID-19 in public health policy and interventions: (i) given the fact that there are a few vaccines, e.g., Pfizer-BioNTech COVID-19, Moderna’s COVID-19 and Oxford/AstraZeneca, available to combat COVID in the UK and internationally at the time of writing, and others are in progress, subject to approvals from both governments and regulatory agencies (MHRA, FDA), and (ii) there have been limited robust published studies of NPIs/SDMs success factors. This scarcity of empirical studies demonstrates the practical realities, e.g., factors or outcomes of NPI would be appropriate for policy planners, researchers and decision-makers to make them effective.

### Strengths and Limitations

To our knowledge, this is the first systematic review (SR) to examine the factors associated with the implementation of NPIs to reduce transmission of COVID-19. It used a systematic and rigorous search strategy to develop an SR protocol. This study also highlighted the themes from the interpretative synthesis and relative contribution of each study (Table S1). This study has also recognised that the effectiveness of NPIs will depend on the credibility of public health authorities, and on strong leadership and commitment from political leaders and institutions.

Several factors limited the present study. First, as it was not externally funded, and therefore time and resource were constrained and the study was unable to include other NPIs (e.g., school closure, closure of childcare facilities, bans on public transport, reducing travel, contact number reduction, work from home, cancellation of public gatherings) or to review grey literature. Second, identified studies are variable in sample size, quality and study population. Most of them had some methodological weaknesses and were open to bias, and the heterogeneity of data precludes a meaningful meta-analysis to measure the impact of specific enablers or barriers, therefore, the findings warrant generalisation. Third, despite the overall satisfactory methodological quality of the included papers, methodologies were poorly reported (mostly those preprint postings in medRxiv), lacking comprehensive strategies for sampling and procedures, and lacking detail in data gathering and analysis, including identifying and dealing with possible confounding factors (Table 2).

Wolkewitz and Puljak [112] further warned that: “there are many methodological challenges related to producing, gathering, analysing, reporting and publishing data in condensed timelines required during a pandemic”. Finally, searching “social distancing” in different databases produced no results. We noticed that the problem of searching for SDMs and COVID-19 studies was mainly due to rapidly-growing COVID-19 studies in PubMed and other search interfaces, which are not visible in the major search databases (PubMed, EMBASE) due to (i) indexing, and (ii) often bibliographic databases failed to capture preprint and unpublished studies including registered clinical trials [113,114], and the majority are commentaries, news, perspectives or opinions [112]. Though Shokraneh [113] provided some useful links specific to COVID-19 resources, still we found this difficult and time-consuming, and the systematic search strategies noted lack of specificity. Additionally, there have been limited robust published studies of NPI success factors, with most studies exploring the process rather than hard or tangible outcomes.

Despite these limitations, this study has the following contributions and future implications. First, in the absence of COVID-19 vaccines globally, public health measures (called NPIs) should be promoted with the aim of reducing contact rates in the population which would help to ultimately reduce the transmission of the virus. Second, while analysing the factors, this study revealed that the effectiveness of a single NPI intervention, implementing in isolation, is likely to be limited, therefore, the combined effect of major NPIs, e.g., SD, quarantine and isolation would be more effective if they are appropriately integrated with enhanced personal hygiene, environmental sanitation and adequate and appropriate use of PPE (use of masks, handwashing, coughing etiquette) as (Anderson et al. [4], p. 933) emphasise that: “individual behaviour will be crucial to control the spread of COVID-19. Personal, rather than government action, in western democracies might be the most important issue. Early self-isolation, seeking medical advice remotely unless symptoms are severe, and social distancing are key.” Third, the findings of this research have answered the research objectives, which were to systematically gather and synthesise the evidence around NPIs, and the factors associated with reducing COVID-19. These objectives were achieved by demonstrating the aspects of measure or findings in 33 unique studies (Table 2 and Table S1). In that sense, this review has provided useful information (enablers and barriers) to researchers, practitioners, policy planners and decision-makers in terms of selecting appropriate measures to promote health and wellbeing by reducing potential risk behaviours. Fourth, the outcome of this review has releveled several policies and pragmatic implications and the potential benefits can be summarised into two parts: (a) this will help public health professionals or healthcare practitioners to better understand the issues related to barriers and enablers associated with controlling COVID-19, and (b), this will help policy-planners, decision-makers and researchers understand the conditions or factors that may facilitate or constrain NPIs, so that they would be able to implement NPI policies and strategies more effectively in the context of primary or community healthcare settings. Fifth, in order to tackle health inequalities during a pandemic, “governments should have developed a bespoke and sophisticated response to building trust among BAME populations in lower socio-economic groups, codesigning culturally competent messaging with communities.” This would have enabled a dialogue and materials that would have ultimately supported not only the adoption of NPIs, but also much higher rates of vaccine uptake [115].

Finally, this would also contribute to developing appropriate policies, guidance and initiatives within the context of other factors, e.g., communication, education, news and social media, culture, inequalities and ethnicities to confirm or extend the significance of NPIs to establish when, how and where fits best for controlling COVID-19, given the fact that there are a few vaccines available to combat COVID in the UK and internationally at the time of writing. 

## 5. Conclusions

Our SR with a large sample size showed the importance of NPIs for reducing COVID-19 infection in the context of global uncertainty. NPIs are a complex form of intervention, with unique enablers and barriers while implementing them at facility levels within a healthcare system. This study has identified that evidence also signals that implementing NPIs, e.g., SD, is generally effective and one of the best ways for preventing or reducing transmission. This study, however, suggests that the effectiveness of any NPIs in isolation is likely to be limited, therefore, a combination of multiple measures, e.g., SD, isolation and quarantine, and workplace distancing, appeared more effective in reducing COVID-19. In addition to this, both the government and the general public should follow test, track, trace and treat policies as well as other public health measures, including physical distancing and the use of face masks and sanitisers for safety. The study concludes that targeted approaches alongside SD might be the way forward, and more acceptable. Further research to promote country- and context-specific adoption of NPIs (e.g., socio-economic, political, cultural, better fact-based communication and behavioural aspects of populations and societies) to deliver public health preventive measures at the primary healthcare level is needed. Furthermore, research comparing the effectiveness of major interventions, e.g., SD, isolation and quarantine and strategies such as strict containment (suppression) and mitigation will help to provide us with more evidence for future pandemics.

## Figures and Tables

**Table 1:** Search strategy used for MEDLINE.

Search Terms	Search Date	Reviewers
**#**1: “COVID 19”(MeSH Terms) OR “COVID 19”(All Fields) OR “sars cov 2”(All Fields) OR “sars cov 2”(MeSH Terms) OR “severe acute respiratory syndrome coronavirus 2”(All Fields) OR “ncov”(All Fields) OR “2019 ncov”(All Fields) OR “coronavirus infections”(MeSH Terms) OR “coronavirus”(MeSH Terms) OR “coronavirus”(All Fields) OR “coronaviruses”(All Fields) OR “betacoronavirus”(MeSH Terms) OR “betacoronavirus”(All Fields) OR “betacoronaviruses”(All Fields) OR “wuhan coronavirus”(All Fields)	12 March 2021	K.R., C.M.L.
**#**2: “social distance”(All Fields) OR “social distancing”(All Fields) OR “cohorting”(All Fields) OR “community containment”(All Fields) OR “isolation strategy”(All Fields) OR “isolation”(All Fields) OR “patient isolation”(All Fields) OR “patient isolation”(MeSH Terms) OR “patient isolators”(All Fields) OR “patient isolators”(MeSH Terms) OR “physical contact”(All Fields) OR “physical distancing”(All Fields) OR “quarantine”(All Fields) OR “quarantines”(All Fields) OR “quarantine”(MeSH Terms) OR OR “quarantined”(All Fields) OR “quarantining”(All Fields) OR “Banning”(All Fields) OR “distancing”(All Fields)		
**#**3: “reduce”(All Fields) OR “reduced”(All Fields) OR “reduces”(All Fields) OR “reducing”(All Fields) OR “transmission”(MeSH Subheading) OR “transmission”(All Fields) OR “transmissions”(All Fields) OR “prevention and control”(MeSH Subheading) OR “prevention and control”(All Fields) OR “prevention”(All Fields) OR “reduce infection”(All Fields)		
**#**4: **#**1 AND **#**2 AND **#**3		

Note: These search terms were modified as needed for use in other databases.

**Table 2:** Summary of selected studies.

Study ID	Aims/Study Question	Country	Type of Study	Samples	JBI Appraisal Tools *					Reviewer Comments
					Number of Questioned Answered				JBI	
					Yes	No	Unclear	NA		
Atchison et al. [65]	To examine risk perceptions and behavioural responses of the UK adult population during the early phase of the COVID-19 epidemic.	UK	Cross-sectional survey	2108	1, 2, 4, 7, 8	3, 5, 8	0	0	Quant. 5/8	Lack of methodological details but plausible analysis.
Cowling et al. [66]	To examine the effect of these interventions and behavioural changes of the public on the incidence of COVID-19, as well as on influenza virus infections, which might share some aspects of transmission dynamics with COVID-19.	Hong Kong	Cross-sectional telephone survey	3013	1, 2, 4, 7, 8	3, 5, 6	0	0	Quant. 5/8	Lack of methodological details but plausible analysis.
Pan et al. [67]	To evaluate the association of public health interventions with the epidemiological features of the COVID-19 outbreak in Wuhan by 5 periods according to key events and interventions.	China	Quantitative survey	32,583	2, 4, 7, 8	1, 3, 5, 6	0	0	Quant. 4/8	Poor methodological details.
Rios-González [68]	To examine the knowledge, attitudes and practices of the population about COVID-19.	Paraguay	Cross-sectional study	3141	2, 3, 7, 8	1, 4, 5, 6	0	0	Quant. 4/8	Poor methodological details.
Roy et al. [69]	To assess the knowledge, attitude, anxiety experience, and perceived mental healthcare need among the adult Indian population during the COVID-19 pandemic.	India	Cross-sectional, observational study	662	1, 2, 7, 8	3, 4, 5, 6	0	0	Quant. 4/8	Poor methodological details.
Al-Hanawi et al. [70]	To investigate COVID-19 knowledge, attitudes and practices (KAP), and associated sociodemographic characteristics among the general population.	Saudi Arabia	Cross-sectional study	3388	1, 2, 3, 4, 6, 7, 8	5	0	0	Quant. 7/8	Few gaps in methodological details but plausible analysis.
Liu et al. [71]	To examine the protective effects of appropriate personal protective equipment for frontline healthcare professionals who provided care for patients with coronavirus disease 2019 (COVID-19).	China	Cross-sectional study	420	1, 2, 3, 4, 6, 7, 8	5	0	0	Quant. 7/8	Few gaps in methodological details but plausible analysis.
Wu et al. [72]	To determine the rate of secondary infection among contacts of individuals with confirmed COVID-19 in Hangzhou according to the type of contact, the intensity of the contact, and their relationship with the index patient.	China	Retrospective cohort study	2994	1, 2, 3, 6, 7, 11	4, 5, 8, 9, 10	0	0	Cohor.7/11	Some gaps in the methodology.
Alobuia et al. [73]	To determine whether disparities exist in the levels of knowledge, attitudes and practices (KAPs) related to COVID-19.	USA	Cross-sectional study	1216	1, 2, 4, 8	3, 5, 6, 7	0	0	Quant. 4/8	Poor methodological details; some gaps in the methodology.
Feng et al. [74]	To explore the influence of altruism on negative affect and mental health (anxiety and depressive symptoms) during the COVID-19 pandemic while people self-isolated at home in China.	China	Cross-sectional study	1346	1, 2, 4, 7, 8	3, 5, 6	0	0	Quant. 5/8	Some gaps in methodology but overall convincing.
Shorey et al. [75]	To analyse the comments left on local media news outlets to find common concerns and discuss potential new measures that can be developed to reduce panic and support for Singapore’s public during and beyond COVID-19.	Singapore	Qualitative study	Not provided (NP)	3, 4, 5, 8, 9, 10	1, 2, 6, 7	0	0	Quali. 6/10	Some gaps in methodology.
Sikkema et al. [76]	To understand sources and modes of transmission of SARS-CoV-2 in healthcare workers and patients.	Netherlands	Cross-sectional study	96	1, 2, 3, 4, 7	5, 6, 8	0	0	Quant. 5/8	Some gaps in methodology but overall convincing.
Rugarabamu et al. [77]	To investigate KAP towards COVID-19 KAP among residents in Tanzania during the April–May 2020 period of the epidemic.	Tanzania	Cross-sectional study	400	2, 4, 7, 8	1, 3, 5, 6	0	0	Quant. 4/8	Some gaps in the methodology.
Grannell et al. [78]	To examine the impact of the pandemic on their lived experience from a treatment and psychosocial standpoint and additionally explore their awareness of obesity as a risk factor for COVID-19 disease severity.	Ireland	Qualitative study	23	1, 2, 3, 4, 5, 8, 9, 10	6, 7	0	0	Quali. 8/10	Some gaps in methodology but overall convincing.
Moorthy and Sankar [79]	To explore the beliefs and perception about the reported worrying issue among the BAME health workforce in the diverse city of Leicester.	UK	Cross-sectional study	200	1, 2, 3, 4, 5, 7, 8	6	0	0	Quant. 7/8	Some gaps in methodological details but plausible analysis.
Solerte et al. [80]	To report several clinical and biochemical outcomes in patients with type 2 diabetes hospitalized for COVID-19.	Italy	Case-control, retrospective study	338	1, 2, 3, 4, 5, 8, 10	6, 7, 9	0	0	Case-Contr. 7/10	Appropriate methodological details and plausible analysis.
Vally [81]	To examine the public’s perceptions of the pandemic, assesses the extent to which participants have adhered to a range of recommended health-protective behaviours to prevent infection and evaluates whether anxiety about COVID-19 or perceptions related to the pandemic are associated with greater adherence to these behaviours.	United Arab Emirates, Abu Dhabi and Dubai	Cross-sectional study	634	1, 2, 3, 4, 7, 8	5, 6	0	0	Quant. 6/8	Some gaps in methodological details but plausible analysis.
Smith et al. [82]	To investigate factors associated with adherence to self-isolation and lockdown measures due to COVID-19 in the UK.	UK	Cross-sectional survey	217	1, 2, 3, 4, 6, 7, 8	5	0	0	Quant. 7/8	Appropriate methodological details and plausible analysis.
Jing et al. [83]	To estimate the secondary attack rate of SARS-CoV-2 among household and non-household close contacts in Guangzhou, China.	China	Retrospective cohort study	349	1, 2, 3, 6, 7, 11	4, 5, 8, 9, 10	0	0	Cohort. 6/11	Some gaps in methodological details but plausible analysis.
Islam et al. [84]	To investigate the KAP toward COVID-19 among slum dwellers resided in Dhaka City, Bangladesh.	Bangladesh	Cross-sectional study	406	1, 2, 3, 4, 7, 8	5, 6	0	0	Quant. 6/8	Some gaps in methodological details but plausible analysis.
Makhashvili et al. [85]	To examine concern about COVID-19 and its association with symptoms of mental disorders in the Republic of Georgia.	Georgia	Cross-sectional study	2088	1, 2, 3, 4, 6, 7, 8	5	0	0	Quant. 7/8	Appropriate methodological details and plausible analysis.
Bäuerle et al. [86]	To assess initial data on the mental health burden of the German public during the COVID-19 pandemic.	Germany	Cross-sectional study	15,037	1, 2, 3, 4, 7, 8	5, 6	0	0	Quant. 6/8	Some gaps in methodology but overall convincing.
Skoda et al. [87]	To close the research gap and provide initial findings on the psychological burden of German healthcare professionals after the COVID-19 outbreak.	Germany	Cross-sectional study	12,863	1, 2, 3, 7, 8	4, 5, 6	0	0	Quant. 5/8	Poor methodological details; some gaps in the methodologies but overall convincing.
Williams et al. [88]	To explore the perceptions and experiences of the UK public of social distancing and social isolation measures related to the COVID-19 pandemic.	UK	Qualitative—focus group study	27	1, 2, 3, 4, 5, 8, 9, 10	6, 7	0	0	Quali. 8/10	Some gaps in methodology but overall convincing.
Mohamed et al. [89]	To assess the knowledge, attitude, and practices of the Sudanese population towards COVID-19.	Sudan	Descriptive cross-sectional	987	1, 2, 3, 4, 7, 8	5, 6	0	0	Quant. 6/8	Some gaps in methodology but overall convincing.
Singh et al. [90]	To explore community perceptions of COVID-19 and their experiences towards health services utilization during the pandemic in Province-2 of Nepal.	Nepal	Qualitative study	41	2, 3, 4, 5, 6, 8, 9, 10	1, 7	0	0	Quali. 8/10	Few gaps in methodological details but plausible analysis.
Wang et al. [91]	To investigate psychological distress among parents of children with ASD during the COVID-19 pandemic.	China	Cross-sectional study	6726	1, 2, 3, 4, 7, 8	5, 6	0	0	Quant. 6/8	Some gaps in methodology but overall convincing.
Wolf et al. [92]	To determine COVID-19 awareness, knowledge, attitudes, and related behaviours among U.S. adults who are more vulnerable to complications of infection because of age and comorbid conditions.	USA	Cross-sectional survey	630	1, 2, 3, 4, 7, 8	5, 6	0	0	Quant. 6/8	Few gaps in methodological details but plausible analysis.
Zhong et al. [93]	To investigate Chinese residents’ KAP towards COVID-19 during the rapid rise period of the outbreak.	China	Cross-sectional survey	6919	1, 2, 3, 4, 7, 8	5.6	0	0	Quant. 6/8	Some gaps in methodology but overall convincing.
Gallè et al. [94]	To (i) evaluate the level of knowledge about the 2019-nCoV, it’s spread and the control measures adopted; (ii) analyse health-related behaviours during lockdown, in order to estimate its possible impact on personal habits; (iii) understand if the study field may influence the level of knowledge and lifestyle habits during the pandemic.	Italy	Quantitative survey	2125	1, 3, 7, 8	2, 4, 5, 6	0	0	Quant. 4/8	Poor methodological details.
Geldsetzer [95]	To assess knowledge and perceptions about COVID-19 among a convenience sample of the general public in the United States and the United Kingdom.	UK and USA	Cross-sectional survey	5974	2, 3, 4, 7, 8	1, 5, 6	0	0	Quant. 5/8	Poor methodological details.
Katz et al. [96]	To identify key features of preparedness and the primary concerns of local public health officials in deciding to implement social distancing measures, and determine whether any particular factor could explain the widespread variation among health departments in responses to past outbreaks.	USA	Cross-sectional online survey	150	1, 2, 4, 7, 8	3, 5, 6	0	0	Quant. 5/8	Lack of methodological details but plausible analysis.
Meier et al. [97]	To evaluate public belief in the effectiveness of protective measures, to what extent individuals have implemented these measures in their daily lives, and to identify key communication channels used to acquire information on COVID-19 in European countries.	Netherlands, Germany and Italy	Cross-sectional survey study	9796	1, 2, 3, 4, 7, 8	5, 6	0	0	Quant. 6/8	Some gaps in methodological details but plausible analysis.

* Numbers in this column signify the quality criteria from the critical appraisal checklist (Table S1) that studies were deemed to have met.

## Supplementary Materials

The following are available online at https://www.mdpi.com/article/10.3390/ijerph18084274/s1, File S1: JBI critical appraisal tools, Table S1: contribution of each study in a thematic synthesis.

## References

[1] World Health Organization WHO Coronavirus (COVID-19) Dashboard..

[2] Chen H., Shi L., Zhang Y., Wang X., Sun G. (2021). A cross-country core strategy comparison in China, Japan, Singapore and South Korea during the early COVID-19 pandemic. Glob. Health.

[3] Alanezi F., Aljahdali A., Alyousef S.M., Alrashed H., Mushcab H., AlThani B., Alghamedy F., Alotaibi H., Saadah A., Alanzi T. (2020). A Comparative Study on the Strategies Adopted by the United Kingdom, India, China, Italy, and Saudi Arabia to Contain the Spread of the COVID-19 Pandemic. J. Healthc. Leadersh..

[4] Anderson R.M., Heesterbeek H., Klinkenberg D., Hollingsworth T.D. (2020). How will country-based mitigation measures influence the course of the COVID-19 epidemic?. Lancet.

[5] Ferguson N., Laydon D., Nedjati-Gilani G., Imai N., Ainslie K., Baguelin M., Bhatia S., Boonyasiri A., Cucunubá Z., Cuomo-Dannenburg G. Report 9-Impact of Non-Pharmaceutical Interventions (NPIs) to Reduce COVID-19 Mortality and Healthcare Demand. https://www.imperial.ac.uk/mrc-global-infectious-disease-analysis/covid-19/report-9-impact-of-npis-on-covid-19/.

[6] Li Z., Chen Q., Feng L., Rodewald L., Xia Y., Yu H., Zhang R., An Z., Yin W., Chen W. (2020). Active case finding with case management: The key to tackling the COVID-19 pandemic. Lancet.

[7] World Health Organization Overview of Public Health and Social Measures in the Context of COVID-19: Interim Guidance. https://apps.who.int/iris/handle/10665/332115.

[8] Ferguson N.M., Cummings D.A.T., Fraser C., Cajka J.C., Cooley P.C., Burke D.S. (2006). Strategies for mitigating an influenza pandemic. Nature.

[9] Lai S., Ruktanonchai N.W., Zhou L., Prosper O., Luo W., Floyd J.R., Wesolowski A., Santillana M., Zhang C., Du X. (2020). Effect of non-pharmaceutical interventions to contain COVID-19 in China. Nature.

[10] Centers for Disease Control and Prevention (2020). Nonpharmaceutical Interventions (NPIs). https://www.cdc.gov/nonpharmaceutical-interventions/index.html.

[11] European Centre for Disease Prevention and Control Coronavirus Disease 2019 (COVID-19) in the EU/EEA and the UK. https://www.ecdc.europa.eu/sites/default/files/documents/covid-19-rapid-risk-assessment-coronavirus-disease-2019-eighth-update-8-april-2020.pdf.

[12] Mal P., Suneel P., Shomeeta P. (2020). Social distancing: A non-pharmacological intervention for COVID-19. J. Pak. Med. Assoc..

[13] Johnson C.Y., SLFA (2020). Social Distancing Could Buy U.S. Valuable Time against Coronavirus..

[14] Kähler C.J., Hain R. (2020). Fundamental protective mechanisms of face masks against droplet infections. J. Aerosol Sci..

[15] World Health Organization COVID-19. https://web.archive.org/web/20200325084602/https://www.who.int/docs/default-source/coronaviruse/transcripts/who-audio-emergencies-coronavirus-press-conference-full-20mar2020.pdf?sfvrsn=1eafbff_0.

[16] Rocklöv J., Sjödin H., Wilder-Smith A. (2020). COVID-19 outbreak on the Diamond Princess cruise ship: Estimating the epidemic potential and effectiveness of public health countermeasure. J. Travel Med..

[17] World Health Organisation Basic Protective Measures against the New Coronavirus. https://www.who.int/emergencies/diseases/novel-coronavirus-2019/advice-for-public.

[18] Holmes R. (2020). Is COVID-19 Social Media’s Levelling Up Moment?. https://www.forbes.com/sites/ryanholmes/2020/04/24/is-covid-19-social-medias-levelling-up-moment/#32e022256c60.

[19] Nabity-Grover T., Cheung C.M.K., Thatcher J.B. (2020). Inside out and outside in: How the COVID-19 pandemic affects self-disclosure on social media. Int. J. Inf. Manag..

[20] Malecki K.M.C., Keating J.A., Safdar N. (2021). Crisis Communication and Public Perception of COVID-19 Risk in the Era of Social Media. Clin. Infect. Dis..

[21] Solnick R.E., Chao G., Ross R.D., Kraft-Todd G.T., Kocher K.E. (2021). Emergency Physicians and Personal Narratives Improve the Perceived Effectiveness of COVID-19 Public Health Recommendations on Social Media: A Randomized Experiment. Acad. Emerg. Med..

[22] Liu Z., Geng H., Chen H., Zhu M., Zhu T. (2020). Exploring the Mechanisms of Influence on COVID-19 Preventive Behaviors in China’s Social Media Users. Int. J. Environ. Res. Public Health.

[23] Oh S.-H., Lee S.Y., Han C. (2020). The Effects of Social Media Use on Preventive Behaviors during Infectious Disease Outbreaks: The Mediating Role of Self-relevant Emotions and Public Risk Perception. Health Commun..

[24] World Health Organization (2020). Social Media & COVID-19: A Global Study of Digital Crisis Interaction among Gen Z and Millennials. https://www.who.int/news-room/feature-stories/detail/social-media-covid-19-a-global-study-of-digital-crisis-interaction-among-gen-z-and-millennials.

[25] Chatwin J., Butler D., Jones J., James L., Choucri L., McCarthy R. (2021). Experiences of pregnant mothers using a social media based antenatal support service during the COVID-19 lockdown in the UK: Findings from a user survey. BMJ Open.

[26] Feng S., Shen C., Xia N., Song W., Fan M., Cowling B.J. (2020). Rational use of face masks in the COVID-19 pandemic. Lancet Respir. Med..

[27] Razaq A., Harrison D., Karunanithi S., Barr B., Asaria M., Khunti K. BAME COVID-19 Deaths-What Do We Know? Rapid Data & Evidence Review: “Hidden in Plain Sight”. https://www.cebm.net/wp-content/uploads/2020/05/BAME-COVID-Rapid-Data-Evidence-Review-Final-Hidden-in-Plain-Sight-compressed.pdf.

[28] Baena-Díez J.M., Barroso M., Cordeiro-Coelho S.I., Díaz J.L., Grau M. (2020). Impact of COVID-19 outbreak by income: Hitting hardest the most deprived. J. Public Health.

[29] Public Health England Disparities in the Risk and Outcomes from COVID-19. https://assets.publishing.service.gov.uk/government/uploads/system/uploads/attachment_data/file/889195/disparities_review.pdf.

[30] van Elsland S.L., O’Hare R. Coronavirus Pandemic Could Have Caused 40 Million Deaths if Left Unchecked. https://www.imperial.ac.uk/news/196496/coronavirus-pandemic-could-have-caused-40/.

[31] Lewnard J.A., Lo N.C. (2020). Scientific and ethical basis for social-distancing interventions against COVID-19. Lancet Infect. Dis..

[32] Marmot M., Allen J., Goldblatt P., Herd E., Morrison J. (2020). Build. Back Fairer: The COVID-19 Marmot Review. The Pandemic, Socioeconomic and Health Inequalities in England.

[33] Haque Z., Becares L., Treloar N. (2020). Over-Exposed and Under-Protected: The Devastating Impact of Covid-19 on Black and Minority Ethnic Communities in Great Britain. https://www.gmcvo.org.uk/news/over-exposed-and-under-protected-devastating-impact-covid-19-black-and-minority-ethnic.

[34] Baker N. (2020). The Housing Pandemic: Four Graphs Showing the Link between COVID-19 Deaths and the Housing Crisis. https://www.insidehousing.co.uk/insight/insight/the-housing-pandemic-four-graphs-showing-the-link-between-covid-19-deaths-and-the-housing-crisis-66562.

[35] Judge L., Rahman F. (2020). Lockdown Living: Housing Quality Across the Generations. https://www.resolutionfoundation.org/publications/lockdown-living/.

[36] Ministry of Housing C & LG (2020). Overcrowded Households. https://www.ethnicity-facts-figures.service.gov.uk/housing/housing-conditions/overcrowded-households/latest.

[37] Butler P. (2020). Poor Housing Linked to High Covid-19 Death Rate in London Borough. https://www.theguardian.com/world/2020/aug/17/poor-housing-linked-high-covid-19-death-rate-london-borough-brent.

[38] Khunti K., Singh A., Pareek M., Hanif W. (2020). Is ethnicity linked to incidence or outcomes of covid-19?. BMJ.

[39] Vandenberg O., Martiny D., Rochas O., van Belkum A., Kozlakidis Z. (2021). Considerations for diagnostic COVID-19 tests. Nat. Rev. Microbiol..

[40] Flaxman S., Mishra S., Gandy A., Unwin H.J.T., Mellan T.A., Coupland H., Whittaker C., Zhu H., Berah T., Eaton J.W. (2020). Estimating the effects of non-pharmaceutical interventions on COVID-19 in Europe. Nature.

[41] Peplow M. (2020). Developing Countries Face Diagnostic Challenges as the COVID-19 Pandemic Surges. https://cen.acs.org/analytical-chemistry/diagnostics/Developing-countries-face-diagnostic-challenges/98/i27.

[42] Park M., Cook A., Lim J., Sun Y., Dickens B. (2020). A Systematic Review of COVID-19 Epidemiology Based on Current Evidence. J. Clin. Med..

[43] Harapan H., Itoh N., Yufika A., Winardi W., Keam S., Te H., Megawati D., Hayati Z., Wagner A.L., Mudatsir M. (2020). The COVID-19 pandemic calls for spatial distancing and social closeness: Not for social distancing!. J. Infect. Public Health.

[44] Viner R.M., Russell S., Croker H., Packer J., Ward J., Stansfield C., Mytton O., Bonell C., Booy R. (2020). School closure and management practices during coronavirus outbreaks including COVID-19: A rapid systematic review. Lancet Child. Adolesc. Health.

[45] Brooks S.K., Webster R.K., Smith L.E., Woodland L., Wessely S., Greenberg N., Rubin G. (2020). The psychological impact of quarantine and how to reduce it: Rapid review of the evidence. Lancet.

[46] Webster R., Brooks S., Smith L., Woodland L., Wessely S., James R. (2020). How to improve adherence with quarantine: Rapid review of the evidence. medRxiv.

[47] Bayham J., Fenichel E. (2020). Impact of school closures for COVID-19 on the US health-care workforce and net mortality: A modelling study. Lancet Public Health.

[48] Hellewell J., Abbott S., Gimma A., Bosse N.I., Jarvis C., Russell T., Munday J., Kucharski A., Edmunds W., Sun F. (2020). Feasibility of controlling COVID-19 outbreaks by isolation of cases and contacts. Lancet Glob. Health.

[49] Prem K., Liu Y., Russell T.W., Kucharski A.J., Eggo R.M., Davies N., Jit M., Klepac P., Flasche S., Clifford S. (2020). The effect of control strategies to reduce social mixing on outcomes of the COVID-19 epidemic in Wuhan, China: A modelling study. Lancet Public Health.

[50] Tang B., Xia F., Tang S., Bragazzi N., Li Q., Sun X., Liang J., Xiao Y., Wu J. (2020). The effectiveness of quarantine and isolation determine the trend of the COVID-19 epidemics in the final phase of the current outbreak in China. Int. J. Infect. Dis..

[51] Zeb A., Alzahrani E., Erturk V.S., Zaman G. (2020). Mathematical Model for Coronavirus Disease 2019 (COVID-19) Containing Isolation Class. Biomed. Res. Int..

[52] Mahtani K.R., Heneghan C., Aronson J.K. What Is the Evidence for social Distancing during Global Pandemics? A Rapid Summary of Current Knowledge. https://www.phc.ox.ac.uk/files/covid-19-evidence-service/what-is-the-evidence-for-social-distancing-during-global-pandemics-final-1.pdf/view.

[53] Rashid H., Ridda I., King C., Begun M., Tekin H., Wood J.G., Booy R. (2015). Evidence compendium and advice on social distancing and other related measures for response to an influenza pandemic. Paediatr. Respir. Rev..

[54] Fong M., Gao H., Wong J., Xiao J., Shiu E., Ryu S., Cowling B. (2020). Nonpharmaceutical measures for pandemic influenza in nonhealthcare settings-social distancing measures. Emerg. Infect. Dis..

[55] Chu D.K., Akl E.A., Duda S., Solo K., Yaacoub S., Schünemann H.J. (2020). Physical distancing, face masks, and eye protection to prevent person-to-person transmission of SARS-CoV-2 and COVID-19: A systematic review and meta-analysis. Lancet.

[56] University of Michigan Michigan Medicine Projections Show Aggressive Social Distancing Will Dramatically Reduce the Peak Number of Hospitalized COVID-19 Patients. https://www.uofmhealth.org/news/archive/202003/michigan-medicine-projections-show-aggressive-social.

[57] Regmi K., Lwin C.M. (2020). Impact of non-pharmaceutical interventions for reducing transmission of COVID-19: A systematic review and meta-analysis protocol. BMJ Open.

[58] Gough D., Oliver S., Thomas J. (2017). An Introduction to Systematic Reviews.

[59] Guyatt G., Rennie D. (2007). Users’ Guides to the Medical Literature: A Manual for Evidenced-Based Clinical Practice.

[60] Borenstein M., Hedges L.V., Higgins J., Rothstein H.R. (2009). Introduction to Meta-Analysis.

[61] Moher D., Liberati A., Tetzlaff J., Altman D.G. (2009). Preferred reporting items for systematic reviews and meta-analyses: The PRISMA statement. BMJ.

[62] Thomas J., Harden A. (2008). Methods for the thematic synthesis of qualitative research in systematic reviews. BMC Med. Res. Methodol..

[63] Moola S., Munn Z., Tufanaru C., Aromataris E., Sears K., Sfetcu R., Currie M., Qureshi R., Mattis P., Lisy K., Aromataris E., Munn Z. (2020). Chapter 7: Systematic reviews of etiology and risk. Joanna Briggs Institute Reviewer’s Manual.

[64] Lockwood C., Munn Z., Porritt K. (2015). Qualitative research synthesis: Methodological guidance for systematic reviewers utilizing meta-aggregation. Int. J. Evid. Based Healthc..

[65] Atchison C., Bowman L., Vrinten C., Redd R., Pristerà P., Eaton J., Ward H. (2021). Early perceptions and behavioural responses during the COVID-19 pandemic: A cross-sectional survey of UK adults. BMJ Open..

[66] Cowling B.J., Ali S.T., Ng T.W.Y., Tsang T.K., Li J.C.M., Fong M.W., Liao Q., Kwan M.Y., Lee S.L., Chiu S.S. (2020). Impact assessment of non-pharmaceutical interventions against coronavirus disease 2019 and influenza in Hong Kong: An observational study. Lancet Public Health.

[67] Pan A., Liu L., Wang C., Guo H., Hao X., Wang Q., Huang J., He N., Yu H., Lin X. (2020). Association of Public Health Interventions With the Epidemiology of the COVID-19 Outbreak in Wuhan, China. JAMA.

[68] Rios-González C.M. (2020). Knowledge, attitudes and practices towards COVID-19 in Paraguayans during outbreaks: A quick online survey. Rev. Salud Publica Parag..

[69] Roy D., Tripathy S., Kar S.K., Sharma N., Verma S.K., Kaushal V. (2020). Study of knowledge, attitude, anxiety & perceived mental healthcare need in Indian population during COVID-19 pandemic. Asian J. Psychiatr..

[70] Al-Hanawi M.K., Angawi K., Alshareef N., Qattan A.M.N., Helmy H.Z., Abudawood Y., Alqurashi M., Kattan W.M., Kadasah N.A., Chirwa G.C. (2020). Knowledge, Attitude and Practice Toward COVID-19 Among the Public in the Kingdom of Saudi Arabia: A Cross-Sectional Study. Front. Public Health.

[71] Liu M., Cheng S.-Z., Xu K.-W., Yang Y., Zhu Q.-T., Zhang H., Yang D.-Y., Cheng S.-Y., Xiao H., Wang J.-W. (2020). Use of personal protective equipment against coronavirus disease 2019 by healthcare professionals in Wuhan, China: Cross sectional study. BMJ.

[72] Wu Y., Song S., Kao Q., Kong Q., Sun Z., Wang B. (2020). Risk of SARS-CoV-2 infection among contacts of individuals with COVID-19 in Hangzhou, China. Public Health.

[73] Alobuia W.M., Dalva-Baird N.P., Forrester J.D., Bendavid E., Bhattacharya J., Kebebew E. (2020). Racial disparities in knowledge, attitudes and practices related to COVID-19 in the USA. J. Public Health.

[74] Feng Y., Zong M., Yang Z., Gu W., Dong D., Qiao Z. (2020). When altruists cannot help: The influence of altruism on the mental health of university students during the COVID-19 pandemic. Global. Health.

[75] Shorey S., Ang E., Yamina A., Tam C. (2020). Perceptions of public on the COVID-19 outbreak in Singapore: A qualitative content analysis. J. Public Health.

[76] Sikkema R.S., Pas S.D., Nieuwenhuijse D.F., O’Toole Á., Verweij J., van der Linden A., Chestakova I., Schapendonk C., Pronk M., Lexmond P. (2020). COVID-19 in health-care workers in three hospitals in the south of the Netherlands: A cross-sectional study. Lancet Infect. Dis..

[77] Rugarabamu S., Ibrahim M., Byanaku A. (2020). Knowledge, attitudes, and practices (KAP) towards COVID-19: A quick online cross-sectional survey among Tanzanian residents. medRxiv.

[78] Grannell A., Roux C.W., McGillicuddy D. (2020). “I am terrified of something happening to me” The lived experience of people with obesity during pandemic. Clin. Obes..

[79] Moorthy A., Sankar T.K. (2020). Emerging public health challenge in UK: Perception and belief on increased COVID19 death among BAME healthcare workers. J. Public Health.

[80] Solerte S.B., D’Addio F., Trevisan R., Lovati E., Rossi A., Pastore I., Dell’Acqua M., Ippolito E., Scaranna C., Bellante R. (2020). Sitagliptin Treatment at the Time of Hospitalization Was Associated With Reduced Mortality in Patients With Type 2 Diabetes and COVID-19: A Multicenter, Case-Control, Retrospective, Observational Study. Diabetes Care.

[81] Vally Z. (2020). Public perceptions, anxiety and the perceived efficacy of health-protective behaviours to mitigate the spread of the SARS-Cov-2/ COVID-19 pandemic. Public Health.

[82] Smith L.E., Amlȏt R., Lambert H., Oliver I., Robin C., Yardley L., Rubin G.J. (2020). Factors associated with adherence to self-isolation and lockdown measures in the UK: A cross-sectional survey. Public Health.

[83] Jing Q.-L., Liu M.-J., Zhang Z.-B., Fang L.-Q., Yuan J., Zhang A.-R., Dean N.E., Luo L., Ma M.-M., Longini I. (2020). Household secondary attack rate of COVID-19 and associated determinants in Guangzhou, China: A retrospective cohort study. Lancet Infect. Dis..

[84] Islam S., Emran G.I., Rahman E., Banik R., Sikder T., Smith L., Hossain S. (2020). Knowledge, attitudes and practices associated with the COVID-19 among slum dwellers resided in Dhaka City: A Bangladeshi interview-based survey. J. Public Health.

[85] Makhashvili N., Javakhishvili J.D., Sturua L., Pilauri K., Fuhr D.C., Roberts B. (2020). The influence of concern about COVID-19 on mental health in the Republic of Georgia: A cross-sectional study. Glob. Health.

[86] Bäuerle A., Teufel M., Musche V., Weismüller B., Kohler H., Hetkamp M., Dörrie N., Schweda A., Skoda E.-M. (2020). Increased generalized anxiety, depression and distress during the COVID-19 pandemic: A cross-sectional study in Germany. J. Public Health.

[87] Skoda E.-M., Teufel M., Stang A., Jöckel K.-H., Junne F., Weismüller B., Hetkamp M., Musche V., Kohler H., Dörrie N. (2020). Psychological burden of healthcare professionals in Germany during the acute phase of the COVID-19 pandemic: Differences and similarities in the international context. J. Public Health.

[88] Williams S., Armitage C., Tampe T., Dienes K. (2020). Public perceptions and experiences of social distancing and social isolation during the COVID-19 pandemic: A UK-based focus group study. BMJ Open.

[89] Mohamed A.A.O., Elhassan E.A.M., Mohamed A.O., Mohammed A.A., Edris H.A., Mahgoop M.A., Sharif M.E., Bashir M.I., Abdelrahim R.B., Idriss W.I. (2021). Knowledge, attitude and practice of the Sudanese people towards COVID-19: An online survey. BMC Public Health.

[90] Singh D.R., Sunuwar D.R., Shah S.K., Karki K., Sah L.K., Adhikari B., Sah R.K. (2021). Impact of COVID-19 on health services utilization in Province-2 of Nepal: A qualitative study among community members and stakeholders. BMC Health Serv. Res..

[91] Wang L., Li D., Pan S., Zhai J., Xia W., Sun C., Zou M. (2021). The relationship between 2019-nCoV and psychological distress among parents of children with autism spectrum disorder. Glob. Health.

[92] Wolf M.S., Serper M., Opsasnick L., O’Conor R.M., Curtis L.M., Benavente J., Wismer G., Batio S., Eifler M. (2020). Awareness, Attitudes, and Actions Related to COVID-19 Among Adults With Chronic Conditions at the Onset of the U.S. Outbreak. Ann. Intern. Med..

[93] Zhong B.L., Luo W., Li H., Zhang Q., Liu X., Li W.T., Li Y. (2020). Knowledge, attitudes, and practices towards COVID-19 among Chinese residents during the rapid rise period of the COVID-19 outbreak: A quick online cross-sectional survey. Int. J. Biol. Sci..

[94] Gallè F., Sabella E., Da Molin G., De Giglio O., Caggiano G., Di Onofrio V., Ferracuti S., Montagna M., Liguori G., Orsi G. (2020). understanding knowledge and behaviors related to CoViD–19 epidemic in Italian undergraduate students: The EPICO Study. Int. J. Environ. Res. Public Health.

[95] Geldsetzer P. (2020). Knowledge and perceptions of covid-19 among the general public in the United States and the United Kingdom: A cross-sectional Online Survey. Ann. Intern. Med..

[96] Katz R., Vaught A., Simmens S. (2019). Local decision making for implementing social distancing in response to outbreaks. Public Health Rep..

[97] Meier K., Glatz T., Guijt M.C., Piccininni M., van der Meulen M., Atmar K., Jolink A.T., Kurth T., Rohmann J.L. (2020). Public perspectives on social distancing and other protective measures in Europe: A cross-sectional 1 survey study during the COVID-19 pandemic. medRxiv.

[98] Hawryluck L., Gold W.L., Robinson S., Pogorski S., Sandro G., Rima S. (2004). SARS control and psychological effects of quarantine, Toronto, Canada. Emerg. Infect. Dis..

[99] Milne G.J., Xie S. (2020). The effectiveness of social distancing in mitigating COVID-19 spread: A modelling analysis. medRxiv.

[100] The Lancet Child & Adolescent Health (2020). Pandemic school closures: Risks and opportunities. Lancet Child. Adolesc. Health.

[101] Sjödin H., Wilder-Smith A., Osman S., Farooq Z., Rocklöv J. (2020). Only strict quarantine measures can curb the coronavirus disease (COVID-19) outbreak in Italy, 2020. Eurosurveillance.

[102] European Centre for Disease Prevention and Control Considerations Relating to Social Distancing Measures in Response to COVID-19–Second Update. https://www.ecdc.europa.eu/en/publications-data/considerations-relating-social-distancing-measures-response-covid-19-second.

[103] Azman A., Luquero F. (2020). From China: Hope and Lessons for COVID-19 Control. Lancet Infect. Dis..

[104] Challen R., Brooks-Pollock E., Read J., Dyson L., Tsaneva-Atanasova K., Danon L. (2021). Risk of mortality in patients infected with SARS-CoV-2 variant of concern 202012/1: Matched cohort study. BMJ.

[105] Martin A., Gravelle T.B., Baekkeskov E., Lewis J., Kashima Y. (2019). Enlisting the support of trusted sources to tackle policy problems: The case of antimicrobial resistance. PLoS ONE.

[106] Gerber A.S., Patashnik E.M., Doherty D., Dowling C.M. (2014). Doctor Knows Best: Physician Endorsements, Public Opinion, and the Politics of Comparative Effectiveness Research. J. Health Polit Policy Law.

[107] Nicol G.E., Piccirillo J.F., Mulsant B.H., Lenze E.J. (2020). Action at a Distance: Geriatric Research during a Pandemic. J. Am. Geriatr. Soc..

[108] Hiremath P., Suhas K., Manjunath M., Shettar M. (2020). COVID 19: Impact of lock-down on mental health and tips to overcome. Asian J. Psychiatr..

[109] Rose T.C., Mason K., Pennington A., Mchale P., Taylor-Robinson D., Barr B. (2020). Inequalities in COVID19 mortality related to ethnicity and socioeconomic deprivation. medRxiv.

[110] Pollock A.M., Roderick P., Cheng K., Pankhania B. (2020). Covid-19: Why is the UK government ignoring WHO’s advice?. Br. Med. J..

[111] Regmi K., Gilbert R., Thunhurst C. (2015). How can health systems be strengthened to control and prevent an Ebola outbreak? A narrative review. Infect. Ecol. Epidemiol..

[112] Wolkewitz M., Puljak L. (2020). Methodological challenges of analysing COVID-19 data during the pandemic. BMC Med. Res. Methodol..

[113] Shokraneh F. (2020). Keeping up with studies on covid-19: Systematic search strategies and resources. Br. Med. J..

[114] Glasziou P. (2020). Waste in covid-19 research. Br. Med. J..

[115] Randhawa G., Griffin S. Covid-19 Must Be a Tipping Point for Tackling Inequalities. https://blogs.bmj.com/bmj/2021/02/12/covid-19-must-be-a-tipping-point-for-tackling-inequalities/.

