# Supplementary material for: Factors Associated with the Implementation of Non-Pharmaceutical Interventions for Reducing Coronavirus Disease 2019 (COVID-19): A Systematic Review
Source: Int J Environ Res Public Health. 2021 Apr 17;18(8):4274. doi: 10.3390/ijerph18084274 (PMC8072790; doi:10.3390/ijerph18084274)
Supplement: Supplementary file 1 [file ijerph-18-04274-s001.zip › ijerph-1174463-SI/File S1. JBI critical appraisal tools.pdf]

File S1: JBI critical appraisal tools

JBI critical appraisal tools for the cross-sectional survey [63], qualitative [64], cohort [63] and case-control [63] studies were used to appraise the retrieved studies with respect to the possibility of biases in their designs, conduct and analysis. The results are provided in Table 1, with number 1-8 (for cross-sectional), 1-10 (for qualitative research), 1-11 (for cohort study) and 1-10 (case-control study) representing satisfactory fulfilment of the corresponding criteria.

Cross-sectional surveys

1) Were the criteria for inclusion in the sample clearly defined?
2) Were the study subjects and the setting described in detail?
3) Was the exposure measured in a valid and reliable way?
4) Were objective, standard criteria used for measurement of the condition?
5) Were confounding factors identified?
6) Were strategies to deal with confounding factors stated?
7) Were the outcomes measured in a valid and reliable way?
8) Was appropriate statistical analysis used?

Qualitative studies

1) Is there congruity between the stated philosophical perspective and the research methodology?
2) Is there congruity between the research methodology and the research question or objectives?
3) Is there congruity between the research methodology and the methods used to collect data?
4) Is there congruity between the research methodology and the representation and analysis of data?
5) Is there congruity between the research methodology and the interpretation of results?
6) Is there a statement locating the researcher culturally or theoretically?
7) Is the influence of the researcher on the research, and vice-versa, addressed?
8) Are participants, and their voices, adequately represented?
9) Is the research ethical according to current criteria, for recent studies, and is there evidence of ethical approval by an appropriate body?
10) Do the conclusions drawn in the research report flow from the analysis, or interpretation, of the data?

Cohort studies

1) Were the two groups similar and recruited from the same population?
2) Were the exposures measured similarly to assign people to both exposed and unexposed groups?
3) Was the exposure measured in a valid and reliable way?
4) Were confounding factors identified?
5) Were strategies to deal with confounding factors stated?
6) Were the groups/participants free of the outcome at the start of the study (or at the moment of exposure)?
7) Were the outcomes measured in a valid and reliable way?
8) Was the follow up time reported and sufficient to be long enough for outcomes to occur?
9) Was follow up complete, and if not, were the reasons to loss to follow up described and explored?
10) Were strategies to address incomplete follow up utilized?
11) Was appropriate statistical analysis used?

Case-control studies

1) Were the groups comparable other than the presence of disease in cases or the absence of disease in controls?
2) Were cases and controls matched appropriately?
3) Were the same criteria used for identification of cases and controls?
4) Was exposure measured in a standard, valid and reliable way?
5) Was exposure measured in the same way for cases and controls?
6) Were confounding factors identified?
7) Were strategies to deal with confounding factors stated?
8) Were outcomes assessed in a standard, valid and reliable way for cases and controls?
9) Was the exposure period of interest long enough to be meaningful?
10) Was appropriate statistical analysis used?
