# Supplementary material for: Factors Associated with the Implementation of Non-Pharmaceutical Interventions for Reducing Coronavirus Disease 2019 (COVID-19): A Systematic Review
Source: Int J Environ Res Public Health. 2021 Apr 17;18(8):4274. doi: 10.3390/ijerph18084274 (PMC8072790; doi:10.3390/ijerph18084274)
Supplement: Supplementary file 1 [file ijerph-18-04274-s001.zip › ijerph-1174463-SI/Table S1. Contribution of each study.pdf]

Table S1: The contribution of each study in a thematic synthesis

	Atchison et al. [65]	Cowling et al. [66]	Pan et al. [67]	Rios-González [68]	Roy et al. [69]	Al-Hanawi et al. [70]	Liu et al. [71]	Wu et al. [72]	Alobuia et al. [73]	Feng et al. [74]	Shorey et al. [75]	Sikkema et al. [76]	Rugarabamu et al. [77]	Gallè et al. [78]	Moorthy & Sankar [79]	Solerte et al. [80]	Vally [81]
▪ Avoiding crowds and social events		√	√		√			√	√	√	√	√		√			√
▪ Reducing reproduction number to avert a local epidemic	√	√	√														
▪ Flatten the peak of and area under the epidemic curve	√	√															
▪ Improve the capacity and capabilities of the healthcare system	√	√		√			√										
▪ Appropriate use of physical measures, e.g. hand sanitizers, handwash and face masks							√		√								√
▪ Border restrictions, school and border closures to suppress transmission		√								√							√
▪ Using multifaceted public health interventions (including border entry restrictions, quarantine and isolation of cases and contacts)	√	√	√	√	√					√	√	√			√		
▪ Changing population behaviour, such as social distancing and personal protective measures		√				√	√	√		√	√	√			√		
▪ Maintain personal hygienic measures					√		√										
▪ Compliance with the government actions	√	√									√			√			
▪ Behavioural changes or interventions which are less disruptive than total lockdown														√			
▪ About the current and second waves or outbreaks		√	√	√				√			√						√
▪ Uncertainty about the duration of the measures								√			√				√		
▪ Lack of trust in governments												√					√
▪ Lack of clarity and understanding				√				√							√		
▪ Not receiving the support expected				√													
▪ Guidance on social distancing and isolation are vague																	
▪ Media constantly giving or reporting wrong information about the pandemic status – resulting in emotional exhaustion					√									√			
▪ Worried after seeing posts about COVID-19 pandemic on various social media platforms					√												

▪ Media affecting mental wellbeing and adding to the level of anxiety					√										√		
▪ Fear and anxiety					√					√							
▪ Increased quarantine associated with post-traumatic stress disorder and depression; decrease of physical activity									√	√				√	√	√	
▪ Loss of social interaction and loss of self-worth					√												
▪ Lack of social and physical contact.<br>▪ Lost job or income and relying on parental, familial or state financial support						√											
▪ COVID-19 associated with ethnic populations in lower socio-economic groups	√		√			√							√		√	√	
▪ Co-morbidity exposure risks and older age ie over 70	√												√			√	

Table S1. The contribution of each study in a thematic synthesis (cont.)

	Smith et al. [82]	Jing et al. [83]	Islam et al. [84]	Makhashvili et al. [85]	Bäuerle et al. [86]	Skoda et al. [87]	Williams et al. [88]	Mohamed et al. [89]	Singh et al. [90]	Wang et al. [91]	Wolf et al. [92]	Zhong et al. [93]	Gallè et al. [94]	Geldsetzer [95]	Katz et al. [96]	Meier et al. [97]
▪ Avoiding crowds and social events					√		√	√	√	√		√	√		√	√
▪ Reducing reproduction number to avert a local epidemic		√														
▪ Flatten the peak of and area under the epidemic curve																
▪ Improve the capacity and capabilities of the healthcare system		√							√		√				√	
▪ Appropriate use of physical measures, e.g. hand sanitizers, handwash and face masks			√		√				√	√						√
▪ Border restrictions, school and border closures to suppress transmission									√							
▪ Using multifaceted public health interventions (including border entry restrictions, quarantine and isolation of cases and contacts)	√	√			√				√		√			√		√
▪ Changing population behaviour, such as social distancing and personal protective measures	√	√							√							
▪ Maintain personal hygienic measures			√						√							√
▪ Compliance with the government actions									√				√			
▪ Behavioural changes or interventions which are less disruptive than total lockdown												√	√			
▪ About the current and second waves or outbreaks							√									√
▪ Uncertainty about the duration of the measures							√		√							
▪ Lack of trust in governments	√				√		√		√							
▪ Lack of clarity and understanding			√													√
▪ Not receiving the support expected																√
▪ Guidance on social distancing and isolation are vague																
▪ Media constantly giving or reporting wrong information about the pandemic status – resulting in emotional exhaustion	√												√			
▪ Worried after seeing posts about COVID-19 pandemic on various social media platforms									√							

▪ Media affecting mental wellbeing and adding to the level of anxiety									√							
▪ Fear and anxiety	√			√	√	√			√	√						
▪ Increased quarantine associated with post-traumatic stress disorder and depression; decrease of physical activity	√		√		√	√							√			
▪ Loss of social interaction and loss of self-worth							√									
▪ Lack of social and physical contact.<br>▪ Lost job or income and relying on parental, familial or state financial support						√	√									
▪ COVID-19 associated with ethnic populations in lower socio-economic groups				√	√		√		√		√			√		
▪ Co-morbidity exposure risks and older age ie over 70											√					

Note: Shading indicates a study was rated as having low quality in terms of the methodological details
